# Intravenous Contrast is Associated with AKI in Patients with Stage 1–3 CKD: PRO

**DOI:** 10.34067/KID.0000000000000186

**Published:** 2023-06-09

**Authors:** Tariku Tadele Gudura, Mohamed Hassanein

**Affiliations:** 1 Department of Kidney Medicine, Cleveland Clinic, Cleveland, Ohio; 2 Department of Nephrology and Hypertension, University of Mississippi Medical Center, Jackson, Mississippi

**Keywords:** clinical nephrology, AKI, CKD

Intravenous (IV) contrast exposure is one of the common causes of AKI among hospitalized patients, accounting for up to 30% of all cases of AKI. The term contrast-induced nephropathy (CIN) was used to describe kidney function decline after contrast media administration within the first 48 hours after excluding other causes of AKI. Owing to the difficulty of differentiating CIN from other nephrotoxic factors causing AKI, the American College of Radiology and the National Kidney Foundation expanded the definition of CIN to Contrast-Induced AKI to describe AKI occurring directly from contrast media exposure signifying a causal relation and Contrast-Associated AKI (CA-AKI) to portray any AKI that occurs after exposure to contrast media, including Contrast-Induced AKI, and is synonymous with the term “Postcontrast AKI.”^1^ CA-AKI has been associated with poor outcomes, including increased cardiovascular morbidity and mortality.^2^ There has been a debate in the literature on the association of IV contrast administration with AKI in earlier stages of CKD. In this article, we provide our “pro” argument favoring the existence of CA-AKI in patients with CKD Stages 1–3.

The underlying mechanisms of kidney injury from IV contrast administration and its causal relation are poorly understood. However, experimental animal studies confirmed that contrast agents decrease kidney blood flow, increase the generation of free oxygen radicals, disrupt mitochondrial membranes, and alter medullary oxygenation because of increased blood viscosity leading to tubular apoptosis, necrosis, and AKI (Figure 1).^3^ Liu *et al.* evaluated the effect of contrast media on afferent and efferent arterioles of mice. Animals exposed to IV iodinated contrast showed a significant reduction of afferent arteriolar diameter from 9.2 to 8.3 *µ*m, whereas it increased from 8.7 to 9.3 *µ*m in the control group. Differential vasoconstriction of the afferent arteriole which is presumed to occur due to decreased nitric oxide production and superoxide generation leads to decreased kidney blood flow.^4^ Mamoulakis *et al.* compared five rabbits exposed to IV iopromide with controls. The mean serum creatinine (SCr) increased by 68.2% within 48 hours in rabbits exposed to contrast compared with no elevation in mean sCr levels in the nonexposed group.^5^ In a uninephrectomized mouse model, water deprivation combined with furosemide and low osmolar contrast media injection caused severe kidney damage and cellular apoptosis, which was not observed without contrast exposure.^6^ In addition, in vitro studies of human embryonic cells by Andreucci *et al.* and cultured proximal tubular cells from study animals by Yang *et al.* confirmed decreased cell proliferation, apoptosis, mitochondrial toxicity, and direct cytotoxicity by iodinated contrast media.^7^

**Figure 1:**
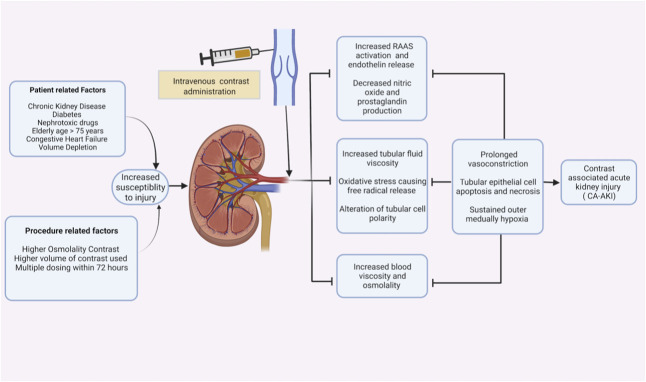
**Proposed mechanisms of CA-AKI.** CA-AKI, contrast-associated AKI; RAAS, renin-angiotensin-aldosterone system. Created with BioRender.com.

Clinical studies have also shown an increased risk of CA-AKI across all stages of CKD, including CKD stages 1–3. Among 12,271 patients who were exposed to IV contrast, Wu *et al.*, reported that CA-AKI occurred across all stages of CKD and increased significantly after CKD stage 3a (*P* < 0.001). Approximately 8.3% of stage 1 (eGFR >90 ml/min per 1.73  m^2^), 6.7% of stage 2 (eGFR 60–90 ml/min per 1.73  m^2^), 9.9% of stage 3a (eGFR 45–60 ml/min per 1.73  m^2^), and 14.3% of stage 3b (eGFR 30–45 ml/min per 1.73  m^2^) developed CA-AKI after exposure to contrast. Moreover, up to 5.7% of patients with CKD stage 1–3 required dialysis within 30 days of contrast exposure.^8^ In a subgroup analysis of 198 patients with oncologic diseases and an eGFR <60 ml/min per 1.73 m^2^, Werner and colleagues found that the incidence of CA-AKI was 4.6% and 7.4% in patients with CKD stage 3a and 3b, respectively.^9^ In a separate analysis of 677 older patients, CA-AKI occurred in 5.9% of patients with eGFR above 90 ml/min per 1.73  m^2^, 9.1% for eGFR 60–90 ml/min per 1.73  m^2^, 7.4% for eGFR 45–60 ml/min per 1.73  m^2^, and 14.9% for eGFR <45 ml/min per 1.73  m^2^, which were all significantly higher than matched controls with similar baseline eGFR. The overall risk of CA-AKI was 1.35 times higher for contrast-exposed patients as compared with controls (*P* < 0.0001), while the relative risk of developing CA-AKI for patients with eGFR <45  ml/min per 1.73  m^2^ compared with those with eGFR >90  ml/min per 1.73  m^2^ was 1.65 (*P* = 0.0043), suggesting higher risk as kidney function declines.^10^ In addition to kidney dysfunction, which is a primary risk factor, other factors that increase the risk of CA-AKI include hypovolemia, heart failure, other nephrotoxins, hypotension, higher contrast media dose, and higher osmolal contrast^11,12^ (Figure 1).

Despite these experimental and observational studies, some studies argue that CA-AKI does not exist, particularly in earlier stages of CKD, or is overreported. However, these studies have major methodological limitations, such as variations in definitions of CA-AKI; lack of uniform dosing of contrast media; lack of adjustment for confounders and effect modifiers, including many patients who are not at higher risk of AKI; lack of a matched control group; the use of prophylactic strategies for prevention of CA-AKI; and selection bias.^6,13^ For example, Heller *et al.* reported no difference in the AKI rate in 6954 patients exposed to IV contrast compared with 909 patients in the control group. However, patients with SCr <1.6 mg/dl were excluded.^14^ More recently, Wilhelm-Leen *et al.* reported no difference in CA-AKI between patients exposed to radiocontrast in a large retrospective nationwide analysis. However, the investigators pointed out that patients who did not receive IV contrast had a higher comorbidity score. In addition, there were no data on baseline kidney function, use of precontrast volume expansion, and volume of contrast used. Moreover, the diagnosis of AKI was based on administrative diagnosis codes rather than actual changes in eGFR.^15^

To sum up, on the basis of animal studies and observational studies in humans, IV contrast is associated with AKI in CKD stages 1–3 and increases significantly after CKD stage 3a. Given the lack of large prospective randomized studies, studies that debate the existence of CA-AKI lack generalizability. As CA-AKI is proven to increase mortality, one has to balance the risks and benefits of using IV contrast agents according to the severity and nature of patient illness and underlying comorbidities.

## Disclosures

All authors have nothing to disclose.
